# Quality Improvement Project on Mental Health (Self-Harm) Care Provision in an Emergency Department

**DOI:** 10.1192/bjo.2025.10359

**Published:** 2025-06-20

**Authors:** Yashar Deylamipour, Jesmine Dhooper, Ekaterina Rykova, Liz Taylor, Abigail Young

**Affiliations:** Dartford and Gravesham NHS Trust, London, United Kingdom

## Abstract

**Aims:** In 2022, the Royal College of Emergency Medicine (RCEM) published an updated toolkit for Mental Health in Emergency Departments (EDs), outlining clinical standards to improve care for mental health patients. These standards, based on guidance from the National Institute for Health and Care Excellence (NICE) and the Royal College of Psychiatrists, focus on (1) the ED mental health triage process, (2) observation of patients at risk of self-harm or absconding, and (3) the quality of ED clinicians’ assessments. The toolkit also emphasises collaboration with mental health teams to facilitate parallel assessments. This quality improvement project evaluated Darent Valley Hospital’s ED performance against these standards and tracked service improvements over two years.

**Methods:** Data was collected retrospectively from October 2022–March 2023 and October 2023–August 2024. A total of 298 cases were analysed (102 in the first year, 196 in the second). Patients aged 18 years and above who presented with intentional self-harm and were referred for an emergency mental health assessment were included. Under 18s, inpatients in mental health units and those not requiring ED care were excluded. Process measures assessed included time to triage, observation of at-risk patients, time to ED clinician review, and risk assessment quality. Outcome measures included indicators of compassionate and practical care, such as provision of food, drink, pain relief and discussions regarding treatment.

**Results:** Monthly meetings with the Psychiatry Liaison Team increased parallel assessments (from 39% to 56%). The appointment of an ED safeguarding lead contributed to reduced times for triage (45 to 40 minutes), and time to physical health assessment (170 to 125 minutes), with dedicated mental health triage compliance increasing (64% to 98%). The proportion of patients receiving well-documented physical health assessments improved from 86% to 92%. While risk assessment quality improved (11% to 17%), particularly regarding drug and alcohol concerns and safeguarding, further work is needed. The presence of alcohol liaison nurses twice weekly supported these improvements. Challenges remain, including a decline in documented observations of at-risk patients (30% to 20%) and only modest improvement in compassionate care provision (13% to 21%).

**Conclusion:** This audit demonstrates progress in assessing and managing patients presenting with self-harm. Planned improvements include a standardised mental health proforma to enhance triage and risk assessment. Further multidisciplinary team discussions will focus on optimising compassionate care, safeguarding, and substance misuse pathways, with ongoing ED staff education. The audit will continue into 2025 to assess the impact of these interventions.

